# Sumoylation of IkB attenuates NF-kB-induced nitrosative stress at rostral ventrolateral medulla and cardiovascular depression in experimental brain death

**DOI:** 10.1186/s12929-016-0283-y

**Published:** 2016-09-22

**Authors:** Ching-Yi Tsai, Faith C. H. Li, Carol H. Y. Wu, Alice Y. W. Chang, Samuel H. H. Chan

**Affiliations:** 1 Institute for Translational Research in Biomedicine, Kaohsiung Chang Gung Memorial Hospital, Kaohsiung, 83301 Taiwan Republic of China; 2 Institute of Physiology, National Cheng Kung University, Tainan, Taiwan Republic of China

**Keywords:** Small ubiquitin-related modifier, Ubiquitin-conjugase 9, Nitrosative stress, Rostral ventrolateral medulla, Defunct brain stem cardiovascular regulation

## Abstract

**Background:**

Small ubiquitin-related modifier (SUMO) is a group of proteins that participates in post-translational modifications. One known SUMO target is the transcription factor nuclear factor-kB (NF-kB) that plays a pivotal role in many disease processes; sumoylation inactivates NF-kB by conjugation with inhibitors of NF-kB (IkB). Our laboratory demonstrated previously that transcriptional upregulation of nitric oxide synthase II (NOS II) by NF-kB, leading to nitrosative stress by the formation of peroxynitrite in the rostral ventrolateral medulla (RVLM), underpins the defunct brain stem cardiovascular regulation that precedes brain death. Based on an experimental endotoxemia model, this study evaluated the hypothesis that sumoylation plays a pro-life role in brain death by interacting with the NF-kB/NOS II/peroxynitrite signaling pathway in the RVLM.

**Results:**

In Sprague–Dawley rats, intravenous administration of *Escherichia coli* lipopolysaccharide (LPS; 10 mg kg^−1^) elicited an augmentation of SUMO-1 and ubiquitin-conjugase 9 (Ubc9) mRNA or protein levels, alongside SUMO-1-conjugated proteins in the RVLM. Immunoneutralization of SUMO-1 or Ubc9 in the RVLM significantly potentiated the already diminished sumoylation of IkBα and intensified NF-kB activation and NOS II/peroxynitrite expression in this brain stem substrate, together with exacerbated fatality, cardiovascular depression and reduction of an experimental index of a life-and-death signal detected from arterial pressure that disappears in comatose patients signifying failure of brain stem cardiovascular regulation before brain death.

**Conclusion:**

We conclude that sumoylation of IkB in the RVLM ameliorates the defunct brain stem cardiovascular regulation that underpins brain death in our experimental endotoxemia modal by reducing nitrosative stress via inhibition of IkB degradation that diminishes the induction of the NF-kB/NOS II/peroxynitrite signaling cascade.

## Background

Small ubiquitin-related modifier (SUMO) is a group of proteins identified about two decades ago [1, 2] that participates in post-translational modifications. Despite the name, SUMO shares only about 18–20 % homology with ubiquitin and is approximately 11 kDa in molecular size [1, 3]. Four SUMO-related proteins, SUMO-1 to SUMO-4, are identified in mammalian cells. SUMO-2 and −3 share 95 % homology with each other, but only 50 % identity with SUMO-1 [4–6]; SUMO-4 shares 87 % amino acid similarity with SUMO-2 [6]. Sumoylation involves the covalent attachment of a member of the SUMO proteins to lysine residues in the target proteins. The process of sumoylation is very similar to ubiquitination and other ubiquitin-like proteins [4], and involves four enzymatic steps: maturation, activation, conjugation and ligation [7]. All SUMO proteins share the same activating (E1) and conjugating (E2) enzymes. Importantly, ubiquitin-conjugase 9 (Ubc9) is the only known E2 enzyme [6, 8].

One of the known SUMO targets is nuclear factor-kB (NF-kB) [9–12], an important transcription factor that regulates many immune and inflammatory genes and plays a pivotal role in lethal endotoxemia [13]. The NF-kB family in mammals consists of five proteins, p65 (RelA), RelB, c-Rel, p50 and p52, which can form transcriptionally active homo- or heterodimers [14, 15]. NF-kB is retained in a latent form in the cytoplasm of non-stimulated cells by inhibitory molecules collectively termed inhibitors of NF-kB (IkB). Stimuli that induce NF-kB activation target IkB to degradation via a phosphorylation- dependent ubiquitination process [16]. Following IkB degradation, NF-kB is translocated to the nucleus as an active transcription factor that is able to induce its target genes [17, 18]. On the other hand, sumoylation can inactivate NF-kB by conjugation with IkB [19, 20]. The sumoylated pool of IkB is protected from ubiquitination, thereby blocking its degradation and inhibits subsequent activation and nuclear translocation of NF-kB [9].

Our laboratory reported previously that one unique prognostic phenotype for brain death exists in the low-frequency (BLF) component (0.04-0.15 Hz in human) of the systemic arterial pressure spectrum [21]. The power density of the BLF component, which reflects the prevailing baroreflex-mediated sympathetic vasomotor tone [22], invariably exhibits a dramatic reduction or loss before brain death ensues in comatose patients who succumbed to systemic inflammatory response syndrome [23], severe brain injury [24] or organophosphate poisoning [25]. We further demonstrated that this life-and-death signal originates from the rostral ventrolateral medulla (RVLM) [26], which is known classically for its role in tonic maintenance of sympathetic vasomotor tone and blood pressure [27]. It follows that this brain stem site is a suitable neural substrate for mechanistic delineation of brain death [21]. In an endotoxemia model of brain death, our laboratory showed previously that the progression towards brain death is causally related to transcriptional upregulation of nitric oxide synthase II (NOS II) induced by NF-kB in the RVLM [28–30]. The eventual fatal culprit is nitrosative stress elicited by the formation of peroxynitrite via a reaction between NOS II-produced nitric oxide (NO) and superoxide anion in the RVLM, of which underpins the loss of BLF power that precedes hypotension and asystole during the progression towards brain death [30, 31].

The present study assessed the guiding hypothesis that sumoylation plays a pro-life role by interacting with the NF-kB/NOS II/peroxynitrite signaling pathway in the RVLM during brain death. Based on an experimental endotoxemia model of brain death, our combined biochemical, physiological and pharmacological results validated this hypothesis. We demonstrated that sumoylation of IkB in the RVLM ameliorates the defunct brain stem cardiovascular regulation during experimental brain death by reducing nitrosative stress via inhibition of IkB degradation that diminishes the induction of the NF-kB/NOS II/peroxynitrite signaling cascade.

## Methods

### Experimental animals

Adult, male Sprague–Dawley rats (202 to 280 g; *n* = 286) purchased from BioLASCO or the Experimental Animal Center of the Ministry of Science and Technology, Taiwan, were used. Animas were housed in an AAALAC International-accredited Center for Laboratory Animals, with maintained room temperature (24 ± 1 °C) and 12-h light–dark cycle (lights on during 07:00–19:00). Animals were allowed to acclimatize for at least 7 days prior to experimental manipulations. Standard laboratory rat chow and tap water were available ad libitum.

### General preparation

Under an induction anesthesia by pentobarbital sodium (50 mg kg^−1^, i.p.), the trachea was intubated and one femoral artery and both femoral veins were cannulated. During the recording session, anesthesia was maintained by intravenous infusion of propofol (Fresenius Kabi, Austria) at 20–25 mg kg^−1^ h^−1^. Based on spectral analysis of arterial pressure (AP) signals and nociceptive tests, our laboratory has demonstrated [32] that this scheme provides satisfactory anesthetic maintenance while preserving the capacity of central cardiovascular regulation. The head of animals was thereafter fixed to a stereotaxic headholder (Kopf, Tujunga, CA), and the body temperature was maintained at 37 °C with a heating pad. During the recording session, animals were allowed to breathe spontaneously with room air.

### Recording and power spectral analysis of systemic arterial pressure signals

AP signals recorded from the femoral artery were processed by an acquisition algorithm at a rate of 1,000 Hz (Notocord, Croissy Sur Seine, France). The digitized signals were analyzed by an arterial blood pressure analyzer (APR31a, Notocord); and were subject to continuous, on-line, and real-time spectral analysis (SPA10a, Notocord) [33, 34]. We were particularly interested in the BLF (0.25-0.8 Hz) band of the AP spectrum for three reasons. First, this spectral component takes origin from the RVLM [26]. Second, it reflects the prevalence of baroreflex-mediated sympathetic vasomotor tone that emanates from this brain stem site [22]. Third and most importantly, the power density of the BLF component represents the crucial link between our animal model and clinical observations from patients who died of systemic inflammatory response syndrome [23], and is a more sensitive prognostic index than AP for brain death [21]. Heart rate (HR) was derived instantaneously from the AP signals.

### Experimental endotoxemia model of brain death

An experimental endotoxemia model of brain death [21], which mimics clinically the progression towards brain death in patients died of systemic inflammatory response syndrome [23] was used. *Escherichia coli* lipopolysaccharide (LPS; 0111:B4 strain; InvivoGen, San Diego, CA) was administrated intravenously at 10 mg kg^−1^ [30], with saline serving as the vehicle control. Temporal changes in pulsatile AP, mean AP (MAP), HR and power density of the BLF component were routinely followed for 300 min, or until the animal succumbed to endotoxemia. The survival rate within 300 min was also recorded.

### Microinjection of test agents into the RVLM

Test agents were microinjected bilaterally and sequentially into the RVLM, at a volume of 50 nL, via a glass micropipette connected to a 0.5-μL Hamilton microsyringe (Reno, NV). The coordinates used were 4.5 to 5 mm posterior to the lambda, 1.8 to 2.1 mm lateral to midline, and 8.1 to 8.4 mm below the dorsal surface of cerebellum [28, 33, 34]. Test agents used in this study included a polyclonal antiserum against SUMO-1 (Cell Signaling Technology, Beverly, MA) generated by immunizing rabbits with a synthetic peptide corresponding to a sequence within SUMO-1 that does not correspond to SUMO-2/3, or a mouse monoclonal antiserum against Ubc9 (BD Biosciences, San Jose, CA). Possible volume effect of microinjection was controlled by injecting the same amount of normal rabbit serum (NRS) or normal mouse serum (NMS) (Sigma-Aldrich, St. Louis, MO). As in our previous studies [34, 35], 0.02 % Triton X-100 (Sigma-Aldrich) was added to facilitate transport of the antiserum, NRS or NMS across the cell membrane of RVLM neurons. All test agents were microinjected bilaterally into the RVLM 30 min before LPS administration. To avoid the confounding effects of drug interactions, each animal received only LPS or saline plus one test agent.

### Collection of tissue samples from the RVLM

We routinely collected tissue samples [29–31] at the peak of each phase of experimental endotoxemia (LPS group), or 20, 150 or 300 min after intravenous injection of saline (saline group). Medullary tissues collected from anesthetized animals but without treatment served as the sham-controls. As a routine, microinjection sites were visually verified and recorded after the slice of medulla oblongata that contains the RVLM (0.5 to 1.5 mm rostral to the obex) was obtained. Tissues from both sides of the ventrolateral medulla were subsequently collected by micropunches made with a 1 mm (i.d.) stainless steel bore to cover the anatomical boundaries of the RVLM. The samples were stored immediately in liquid nitrogen.

### Isolation of RNA and real-time PCR

Total RNA from the RVLM was isolated with a Total RNA Mini kit (Geneaid, Taipei, Taiwan). All RNA isolated was quantified by spectrophotometry and the optical density 260/280 nm ratio was determined. As in our previous studies [34, 36, 37], reverse transcriptase reaction was performed using a Transcriptor First strand cDNA Synthesis kit (Roche, Mannheim, Germany). Real-time PCR analysis was performed by amplification of cDNA using a LightCycler (Roche). Genes were quantified by SYBR Green real-time polymerase chain reaction with *gapdh* as the endogenous control. Primers were designed by Roche LightCycler probe design software 2.0 using the sequence information of the NCBI database, and oligonucleotides were synthesized by Quality Systems (Taipei, Taiwan). The primer pairs used for amplification of the target genes were:

- *sumo1* (Genbank Accession: NM_001009672):
- Forward primer: 5′-TGTCTGACCAGGAGGCA-3′
- Reverse primer: 5′-ACAGTACGATTCTTTGAGCTT-3′
- *ubc9* (Genbank Accession: NM_001180123):
- Forward primer: 5′-TTTCCGCCACTGGCATA-3′
- Reverse primer: 5′-AGCGGACGAGAAGAAACTA-3′
- *gapdh* (Genbank Accession: NM_017008):
- Forward primer: 5′-CTTCTCTTGTGACAAAGTGGA-3′
- Reverse primer: 5′-TTAGCGGGATCTCGCTC-3′

The relative changes in mRNA expression were determined by the fold-change analysis, in which fold change = 2^−[ΔΔCt]^, where ΔΔCt = (Ct_gene_ − Ct_gapdh_) _LPS or saline treatment_ − (Ct_gene_ − Ct_gapdh_) _sham control_). Note that Ct value is the cycle number at which fluorescence signal crosses the threshold.

### Protein extraction and western blot analysis

As in previous studies [33, 34, 37], tissue samples from RVLM were homogenized on ice in a protein extraction buffer that contains protease and phosphatase inhibitors, and centrifuged at 10,000 g at 4 °C for 10 min. The concentration of total proteins was determined by the BCA protein assay (Pierce, Rockford, IL), and absorbance was measured at 562 nm. Western blot analysis was carried out using a rabbit polyclonal antiserum against SUMO-1 (Cell Signaling) or NOS II (Santa Cruz Biotechnology, Santa Cruz, CA); or a mouse monoclonal antiserum against Ubc9 (BD Biosciences), IkBα (Cell Signaling), nitrotyrosine (Upstate, Lake Placid, NY) or β-actin (Chemicon). This was followed by incubation with horseradish peroxidase-conjugated donkey anti-rabbit IgG (GE Healthcare, Little Chalfont, Buckinghamshire, UK), or sheep anti-mouse IgG (GE Healthcare). Specific antibody-antigen complex was detected using an enhanced chemiluminescence western blot detection system (Santa Cruz). The amount of protein expression was quantified by the ChemiDoc XRS^+^ System (Bio-Rad, Hercules, CA), and was expressed as the ratio relative to β-actin protein.

### Immunoprecipitation and immunoblot analysis

Protein extracts from samples of the RVLM were immunoprecipitated with an affinity-purified antiserum against IkBα (Cell Signaling) that was conjugated with protein G-agarose beads (Roche). Immunoprecipitation was performed at 4 °C overnight and the precipitated beads obtained after centrifuged at 10,000 g for 30 s were washed three times with ice-cold lysis buffer. The agarose beads resuspended in the loading buffer were boiled for 10 min. After dissociated from the beads, the immunoprecipitated protein was subjected to immunblot analysis using an anti-rabbit antiserum against SUMO-1 (Cell Signaling).

### Measurement of transcriptional activity of NF-kB p65

Transcriptional activity of NF-kB p65 was measured by a sensitive colorimetric assay (TransAM NF-kB p65; Active Motif; Carlsbad, CA) according to the manufacturer’s protocol. Briefly, nuclear protein extracted from the RVLM was incubated with an immobilized oligonucleotide containing the NF-kB consensus-binding site (5′-GGGACTTTCC-3′). This was followed by incubation with a primary NF-kB p65 antibody, and a secondary peroxidase-conjugated antibody at room temperature. After a colorimetric reaction, the optical density was read at 450 nm using an ELISA microtiter plate reader.

### Statistical analysis

All values are expressed as mean ± SEM. The averaged value of MAP or HR calculated every 20 min after administration of LPS or saline, the sum of power density of BLF component in the AP spectrum over 20 min, and protein expression level or the relative mRNA expression in the RVLM during each phase of experimental endotoxemia, was used for statistical analysis. One-way or two-way analysis of variance with repeated measures was used to assess group means, followed by the Scheffé multiple range test for *post hoc* assessment of individual means. *P <* 0.05 was considered to be statistically significant.

## Results

### Triphasic cardiovascular responses in experimental endotoxemia model of brain death

**Fig. 1:**
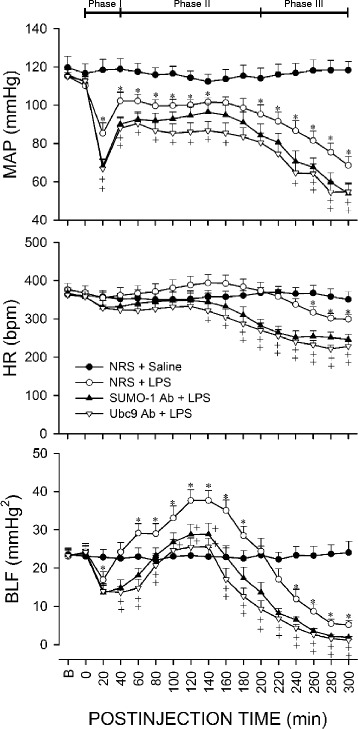
Temporal changes in mean arterial pressure (MAP), heart rate (HR) or power density of the low-frequency (BLF) component of AP spectrum in rats that received pretreatment by microinjection bilaterally into the RVLM of normal rat serum (NRS; 1:20), anti-SUMO-1 antiserum (SUMO-1 Ab; 1:20) or anti-Ubc9 antiserum (Ubc9 Ab; 1:20), followed by intravenous administration of saline or LPS (10 mg kg^−1^). The three distinct phases based on reduced (Phase I), augmented (Phase II) and a secondary decreased (Phase III) power density of the BLF spectral component induced by LPS are denoted on top of the figure. Values are mean ± SEM of 7–8 animals per experimental group. *P < 0.05 versus NRS + saline group, and ^+^ *P* < 0.05 versus NRS + LPS group at corresponding time-points in the post hoc Scheffé multiple-range test. B = preinjection baseline. Note that for clarity of illustration, data on pretreatment with normal mouse serum in this and Figs. 4 to 6 were omitted because they were comparable to those with NRS pretreatment

Figure 1 showed that, similar to our previous studies [29, 30, 36], intravenous administration of LPS (10 mg kg^−1^) in our control animals pretreated with microinjection bilaterally into RVLM of normal serum (1:20) elicited a reduction (Phase I), augmentation (Phase II; pro-life phase) and a secondary decrease (Phase III; pro-death phase) in the power density of the BLF component of AP signals. MAP underwent typically a significant decrease during Phase I, followed by a rebound (Phase II) and progressive hypotension during Phase III. Significant reduction in HR only took place towards the last 40 min of our 300-min observation.

### Upregulation of SUMO-1 and Ubc9 in the RVLM during experimental brain death

**Fig. 2:**
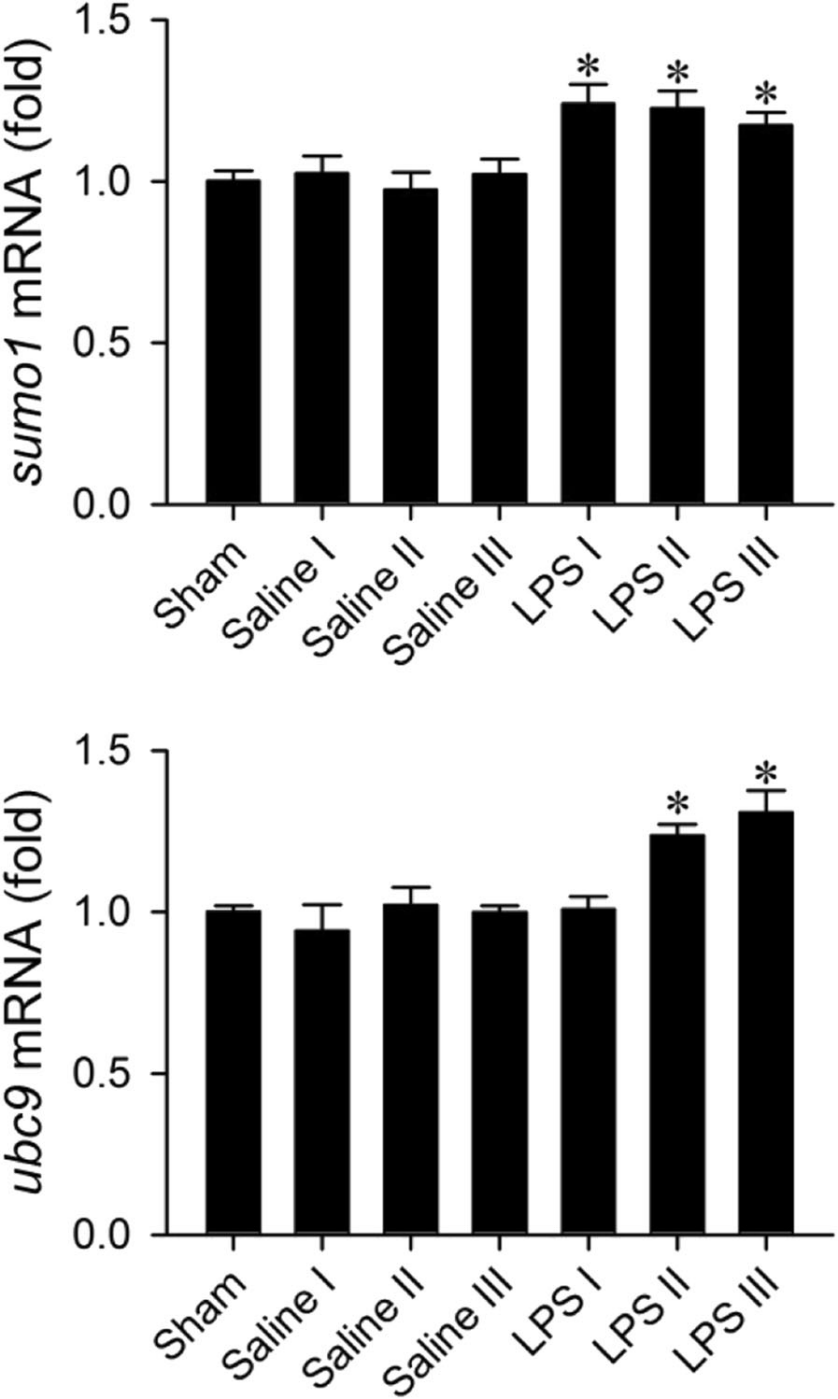
Phasic fold-changes relative to sham-controls of *sumo1* or *ubc9* mRNA in the RVLM determined by real-time PCR after intravenous administration of saline or LPS (10 mg kg^−1^). Tissue samples were collected during the peak of Phases I, II or III in the LPS group; or 20 (I), 150 (II) or 300 (III) min in the saline group. Values are mean ± SEM of triplicate analyses on individual samples obtained from 4–5 animals per experimental group. *P < 0.05 versus sham-control group or corresponding saline group in the post hoc Scheffé multiple-range test

**Fig. 3:**
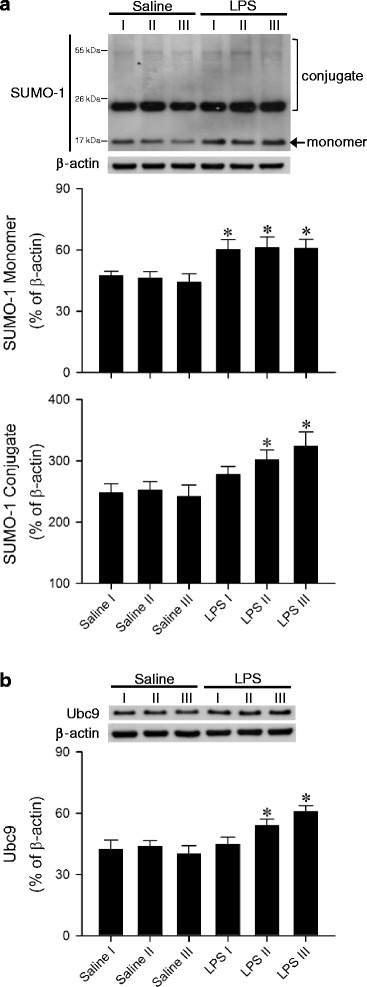
Representative western blots (insets) or percentage of SUMO-1 monomer and SUMO-1 conjugate (**a**) or Ubc9 (**b**) relative to β-actin detected in the RVLM during Phases I, II or III after intravenous administration of saline or LPS (10 mg kg^−1^). Values are mean ± SEM of duplicate analyses on individual samples obtained from 5–6 animals per experimental group. *P < 0.05 versus corresponding saline group in the post hoc Scheffé multiple-range test

Our first series of experiments evaluated the changes of SUMO-1 and Ubc9 in the RVLM during experimental brain death. Real-time PCR analysis revealed that, compared to sham-control and saline-controls, the *sumo1* or *ubc9* mRNA (Fig. 2) level in the RVLM was significantly elevated in our experimental endotoxemia model. Similarly, results from western blot analysis showed significant augmentation of SUMO-1 monomer (Fig. 3a) or Ubc9 (Fig. 3b) protein level in the RVLM. In addition, there was a significant elevation in the amount of SUMO-1-conjugated proteins (Fig. 3a).

### Sumoylation in the RVLM plays a pro-life role during experimental brain death

**Fig. 4:**
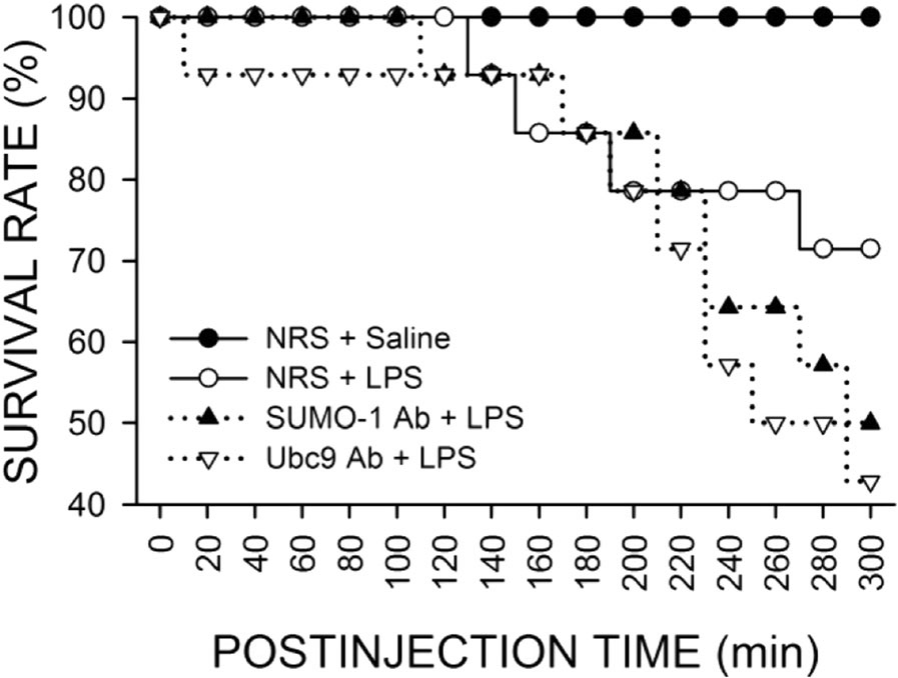
Survival rate over 300 min of animals that received pretreatment by microinjection bilaterally into the RVLM of an antiserum directed against SUMO-1 (SUMO-1 Ab; 1:20) or Ubc9 (Ubc9 Ab; 1:20), or normal rabbit serum (NRS; 1:20), followed by intravenous administration of saline or LPS (10 mg kg^−1^). Each group contained 11–14 animals at the beginning of the experiment

Our second series of experiments employed immunoneutralization to establish a causal relationship between augmented sumoylation and survival rate during experimental brain death. At the dose (10 mg kg^−1^) used, intravenous administration of LPS elicited approximately 28.6 % (4 of 14 animals) fatality within 300 min of administration (Fig. 4). Pretreatment with microinjection into the bilateral RVLM of an anti-SUMO-1 antiserum (1:20) or anti-Ubc9 antiserum (1:20) 30 min before the induction of experimental brain death resulted in 50.0 % (7 of 14 animals) or 57.1 % (8 of 14 animals) death within the same time-period (Fig. 4).

### Sumoylation in the RVLM plays a pro-life role by ameliorating failure of brain stem cardiovascular regulation

Pretreatment by microinjection into the bilateral RVLM of an anti-SUMO-1 or anti-Ubc9 antiserum (Fig. 1) significantly potentiated the elicited hypotension or bradycardia by LPS. The same pretreatment also eliminated the increase in the power density of the BLF component during the pro-life Phase II, and significantly enhanced the reduction in BLF power towards zero level during the pro-death Phase III.

### Conjugation of SUMO-1 with IkBα attenuates NF-kB activation in RVLM during experimental brain death

**Fig. 5:**
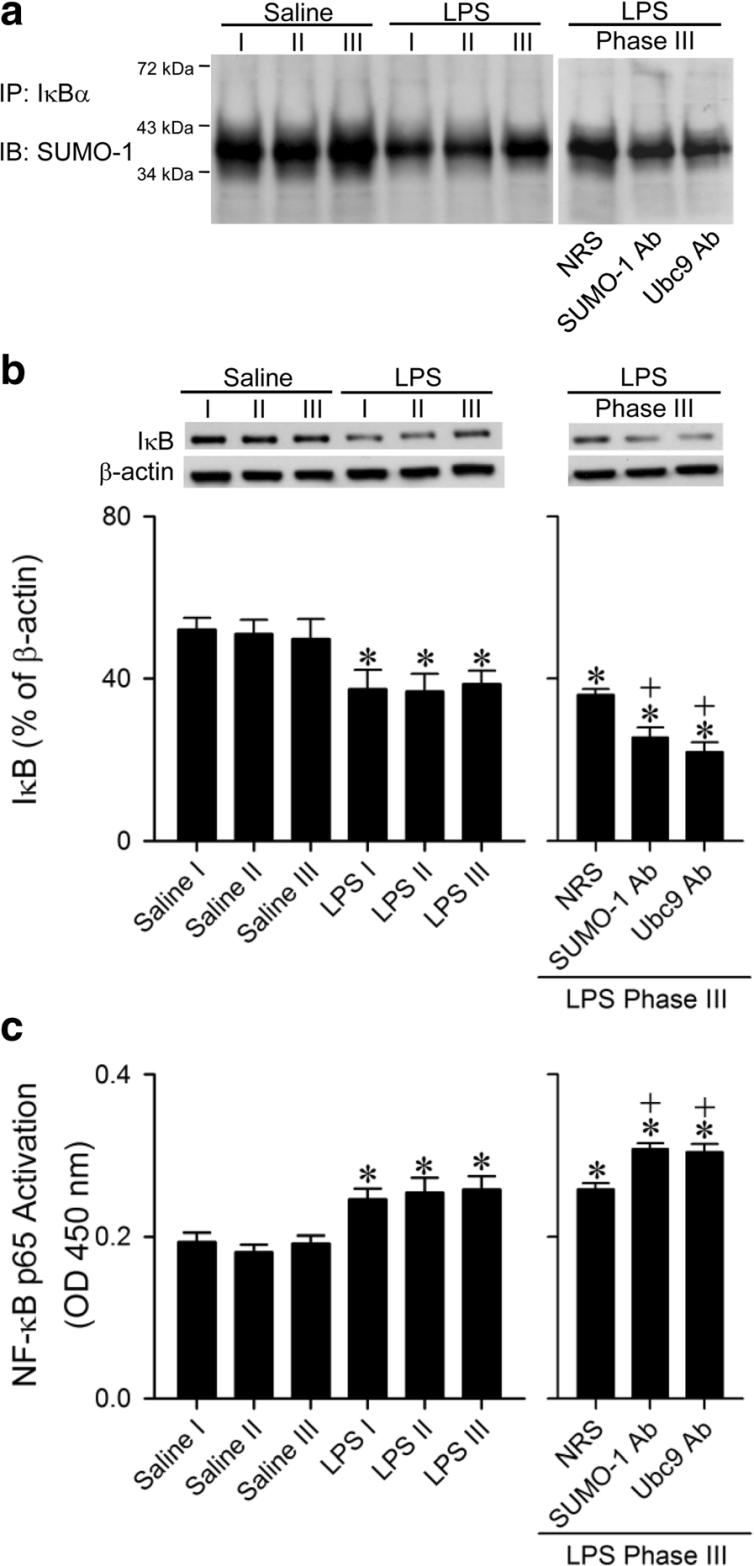
Results from immunoblot (IB) using an antiserum against SUMO-1 on proteins immunoprecipitated (IP) by an anti-IkBα antiserum from the RVLM (**a**); western blot analysis of expression of IkBα relative to β-actin in the RVLM (**b**); transcriptional activity of NF-kB p65 in the RVLM (**c**) after intravenous administration of saline or LPS (10 mg kg^−1^); or animals that received pretreatment by microinjection bilaterally into RVLM of NRS (1:20), anti-SUMO-1 antiserum (SUMO-1 Ab; 1:20) or anti-Ubc9 antiserum (Ubc9 Ab; 1:20) during Phase III in the experimental endotoxemia model of brain death. Values are mean ± SEM of duplicate analyses on individual samples obtained from 5–6 animals per experimental group. *P < 0.05 versus corresponding saline group and ^+^ *P* < 0.05 versus NRS + LPS group in the post hoc Scheffé multiple-range test

We reported previously [30] that activation of NF-kB preferentially upregulates NOS II in the RVLM during experimental brain death. Our fourth series of experiments investigated whether SUMO-1 may impede this cellular process by inactivation of NF-kB via conjugation with IkBα [14–16]. Results from our co-immunoprecipitation and immunoblot analysis (Fig. 5a) showed that the degree of sumoylated IkBα in the RVLM was significantly reduced in our experimental endotoxemia model. Likewise, there was a significant decrease in protein expression of IkBα (Fig. 5b), alongside elevated transcriptional activity of NF-kB p65 in the RVLM (Fig. 5c).

In an attempt to confirm a causal relationship between sumoylation of IkBα and attenuation of NF-kB activation in the RVLM, co-immunoprecipitation experiments (Fig. 5a) during experimental brain death showed that pretreatment with microinjection bilaterally of an anti-SUMO-1 or anti-Ubc9 antiserum (1:20) into the RVLM exacerbated the already diminished sumoylated IkBα. This pretreatment also potentiated the reduction in IkBα protein level (Fig. 5b), and significantly enhanced NF-kB p65 activation (Fig. 5c) in the RVLM.

### Sumoylation reduces nitrosative stress in the RVLM during experimental brain death

**Fig. 6:**
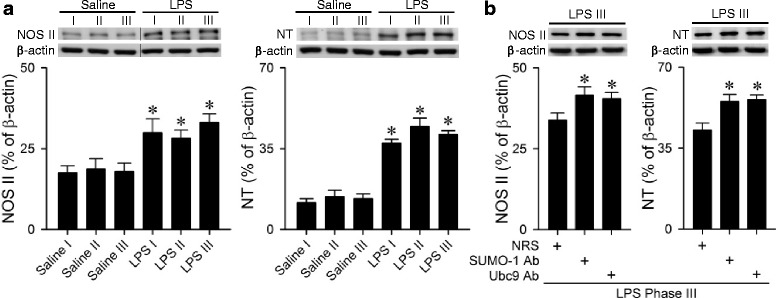
Representative western blots (insets), or percentage of NOS II or nitrotyrosine (NT) relative to β-actin in the RVLM detected after intravenous administration of LPS (10 mg kg^−1^) in animals (**a**); or animals that received pretreatment by microinjection bilaterally into the RVLM of NRS (1:20), anti-SUMO-1 antiserum (SUMO-1 Ab; 1:20) or anti-Ubc9 antiserum (Ubc9 Ab; 1:20) during phase III in the experimental endotoxemia model of brain death (**b**). Values are mean ± SEM of duplicate analyses on individual samples obtained from 5–6 animals per experimental group. *P < 0.05 versus corresponding saline group in the post hoc Scheffé multiple-range test. Note that in (**a**), dividing lines are placed on the representative gel images to denote re-grouping of images from different parts of the same gel

Our final series investigated whether sumoylation reduces the augmented nitrosative stress in the RVLM during experimental brain death [28, 30]. Results from western blot analysis (Fig. 6a) showed that the level of NOS II or nitrotyrosine, an experimental index for peroxynitrite [38], was significantly augmented in our experimental endotoxemia model. Pretreatment with an anti-SUMO-1 or anti-Ubc9 antiserum further potentiated this upregulation of NOS II or nitrotyrosine expression (Fig. 6b) in the RVLM.

## Discussion

Based on an experimental endotoxemia model that mimics clinically comatose patients who succumbed to systemic inflammatory response syndrome [23], the present study provided two intriguing mechanistic insights into brain death. First, sumoylation in the RVLM is augmented during brain death, and one of the cellular targets of SUMO-1 is IkBα. Second, sumoylation of IkBα reduces its degradation, followed by attenuation of transcriptional activation of NOS II synthesis by NF-kB and subsequent formation of peroxynitrite. The resultant diminution of nitrosative stress in the RVLM leads to amelioration of the failure of brain stem cardiovascular regulation that underpins cardiovascular depression during experimental brain death.

SUMO proteins are highly conserved in a large number of species and are engaged in many eukaryotic cell processes [39], including cell cycle regulation, transcription, cellular localization, degradation and chromatin organization [3]. It is well documented that global increases in protein sumoylation occur in response to cellular stress [6, 40–42], and the general consensus is that this results in a protective action against stress. It is therefore of interest that results from the present study assigned a pro-life role to sumoylation at the RVLM in our experimental endotoxemia model of brain death. Based on real-time PCR and western blot analysis, we demonstrated that SUMO-1 and Ubc9 expression at mRNA and protein levels in the RVLM was significantly elevated during experimental brain death. Immunoneutralization of SUMO-1 or Ubc9 increased fatality and significantly potentiated the elicited hypotension or bradycardia. Most importantly, there was significant exacerbation of the already reduced power density of the BLF component towards zero level that is causally related to the manifestation of brain death in a clinical setting [21, 23–25].

An elevation in SUMO-1 conjugation is not necessarily accompanied by SUMO-2/3 conjugation or vice versa. This suggests that sumoylation is controlled in a substrate-specific manner [6]. For example, SUMO-1 modification of IkBα has been observed in Hela, COS7, HEK 293 cell lines [8], rat mesangial cell [43] and CD37 null mouse lung tissue [44]. Immunoprecipitation experiments in the present study also identified IkBα in the RVLM as a conjugation target for SUMO-1. However, despite the elevated amount of SUMO-1 conjugated proteins, we found that sumoylation of IkBα and cytosolic presence of IkBα in the RVLM per se was significantly decreased during experimental brain death. This seeming discrepancy may be resolved by the observation [9, 45, 46] that IkBα is a common cellular target of both sumoylation and ubiquitination. Phosphorylation of IkBα, at serine residues 32 and 36, results in its ubiquitination at lysine residues 21 and 22 and rapid degradation by the proteasome [45, 46]. Desterro et al. [9] reported that SUMO-1 modification of IkBα also takes place at lysine residue 21 and competes with ubiquitin for the same target residue. Sumoylation can inactivate NF-kB by conjugation with IkB [19, 20]; the sumoylated pool of IkB is protected from ubiquitination, thereby blocking its degradation and inhibits subsequent activation and nuclear translocation of NF-kB [9]. Our results showed that whereas the level of IkBα protein in the RVLM was reduced by approximately 26.5 % during experimental brain death, immunoneutralization of SUMO-1 or Ubc9 aggravated these reductions to 51.5 % or 56.6 %. It is therefore conceivable that despite the protection from degradation offered by sumoylation, the tilt of balance in favor of ubiquitination of IkBα accounts for our observed reduction in IkBα protein and sumoylated IkBα in the RVLM during experimental brain death.

Our laboratory demonstrated previously that the progression towards brain death is causally related to transcriptional upregulation of NOS II induced by NF-kB, activated after IkB degradation via ubiquitination in the RVLM [28–30, 47]. The eventual fatal culprit is nitrosative stress elicited by the formation of peroxynitrite via a reaction between NOS II-produced NO and superoxide anion in the RVLM, of which underpins the defunct brain stem cardiovascular regulation that precedes hypotension and asystole during brain death [30, 31]. The present study revealed that sumoylation plays a pro-life role in this process via protecting IkB from degradation by ubiquitination, leading to amelioration of NF-kB activity and the eventual reduction in nitrosative stress in the RVLM in our endotoxemia model of experimental brain death. In addition to protection against IkB degradation, sumoylation may reduce NF-kB activity by direct modification of the NF-kB subunit RelB [48]. Sumoylation is also reported [49] to decrease NOS II expression in LPS-induced inflammatory responses in astrocytes via modification of C/EBPβ at the NOS II promoter. In a parallel study using an organophosphate intoxication model of brain death [47, 50], we demonstrated that SUMO-1 and NF-kB are present in RVLM neurons. We also showed previously that the cellular source of NOS II in the RVLM include neurons, astrocytes and microglia [31]. Together with observations from the present study, it is conceivable that the aforementioned interacting cascades between sumoylation, NF-kB and NOS II may take place in neurons, astrocytes and microglia at the RVLM.

Our laboratory revealed recently [30] that the ubiquitin-proteasome system plays a double-edged sword role in the maintained and defunct brain stem cardiovascular regulation during experimental brain death via the temporal balance between the continuous degradation and progressively augmented synthesis of NOS II. We are cognizant that, against our demonstration that sumoylation of IkBα leads to inactivation of NF-kB, promotion of NF-kB activation in response to genotoxic stress via SUMO-1 modification of NF-kB essential modifier (NEMO, also known as IKKγ) has been demonstrated [51]. We noted from the western blot image in Fig. 3a that among the elevated SUMO-1-conjugated proteins was a conspicuous band around 55 kDa. With a molecular weight of 48 kDa [52], it is possible that sumoylation of NEMO (SUMO-1 monomer has a molecular weight of 11 kDa) also takes place at the RVLM during experimental endotoxemia. Likewise, SUMO-2/3 modification of the same lysine residue 21 and 22 of IkBα, contrary to SUMO-1, induces IkBα degradation and NF-kB activation after TNFα-stimulation [11]. Whether sumoylation similarly plays a double-edged sword role in the regulation of NF-kB/NOS II/peroxynitrite cascade in the RVLM during brain death requires further delineation.

## Conclusions

In conclusion, the present study provided novel findings to support the notion that sumoylation in RVLM plays a pro-life role during experimental brain death. Mechanistically, we found that sumoylation of IkBα ameliorates the defunct brain stem cardiovascular regulation that underpins brain death by reducing nitrosative stress in the RVLM via downregulation of the NF-kB/NOS II/peroxynitrite signaling cascade. In a parallel study using an organophosphate intoxication model of brain death [50], we demonstrated previously that sumoylation exerts its pro-life role via inhibition of hypoxia-inducible factor-1α degradation, leading to upregulation of NOS I/protein kinase G cascade. Brain death, the legal definition of death in many countries [53–56], is a phenomenon of paramount medical importance. Since brain death is an irreversible process, the identification that sumoylation in the RVLM plays a crucial pro-life role in the progression towards brain death by inhibiting the NOS II/peroxynitrite cascade and enhancing the NOS I/protein kinase G signaling offers new insights for devising clinical management or therapeutic strategy against this fatal eventuality.

## Abbreviations

AP: Arterial pressure
BLF: Low-frequency component
HR: Heart rate
IkB: Inhibitors of NF-kB
LPS: Lipopolysaccharide
MAP: Mean arterial pressure
NEMO: NF-kB essential modifier
NF-kB: Nuclear factor-kB
NMS: Normal mouse serum
NO: Nitric oxide
NOS: Nitric oxide synthase
NRS: Normal rabbit serum
RVLM: Rostral ventrolateral medulla
SUMO: Small ubiquitin-related modifier
Ubc9: Ubiquitin-conjugase 9
